# Cancer stem cell‐like characteristics and telomerase activity of the nasopharyngeal carcinoma radioresistant cell line CNE‐2R

**DOI:** 10.1002/cam4.1729

**Published:** 2018-08-13

**Authors:** Kai‐Hua Chen, Ya Guo, Ling Li, Song Qu, Wei Zhao, Qi‐Teng Lu, Qi‐Yan Mo, Bin‐Bin Yu, Lei Zhou, Guo‐Xiang Lin, Yong‐Chu Sun, Xiao‐Dong Zhu

**Affiliations:** ^1^ Department of Radiation Oncology Affiliated Tumor Hospital of Guangxi Medical University Cancer Institute of Guangxi Zhuang Autonomous Region Nanning Guangxi China; ^2^ Department of Radiation Oncology The Second Affiliated Hospital of Xi'an Jiaotong University Xi'an Shanxi China; ^3^ Key Laboratory of Early Prevention and Treatment for Regional High Frequency Tumor (Guangxi Medical University) Ministry of Education Nanning Guangxi China; ^4^ Wuming Hospital of Guangxi Medical University Nanning Guangxi China

**Keywords:** cancer stem cells, nasopharyngeal carcinoma, radioresistance, telomerase activity

## Abstract

The radioresistance of nasopharyngeal carcinoma (NPC) may be related to cancer stem cells (CSCs), and the characteristics of CSCs may be maintained by telomerase activity. In this study, we explored the CSC‐like characteristics and telomerase activity of the NPC radioresistant cell line CNE‐2R. This work provides a foundation for future studies on stem cell‐targeted therapies by targeting the radioresistance of NPC. The expression of stem cell‐related genes/proteins and the hTERT gene/protein in CNE‐2R and its parent radiosensitive cell line CNE‐2 were detected using qPCR/Western Blot. Label‐retaining cells (LRCs) were detected through immunocytochemistry, and telomerase activity was detected using a PCR‐ELISA kit. CD133 expression was detected with flow cytometry. CNE‐2R‐CD133+ and CNE‐2R‐CD133− cells were separated with magnetic‐activated cell sorting. The proliferation and tumorigenesis capacities of CNE‐2R‐CD133+, CNE‐2R‐CD133−, and CNE‐2R cells were compared with a CCK‐8 assay, sphere formation assay, and an in vivo experiment. Our results showed that the expression of stem cell‐related genes and the hTERT gene in CNE‐2R cells was higher than those in CNE‐2 cells. Similarly, the expression of stem cell‐related proteins and the hTERT protein in CNE‐2R cells was markedly higher than those in CNE‐2 cells. The proportion of LRCs in CNE‐2R and CNE‐2 cells was (3.10 ± 0.63%) vs (0.40 ± 0.35%; *P *<* *0.001), respectively. Telomerase activity in CNE‐2R cells was remarkably higher than that in CNE‐2 cells. Flow cytometry suggested that the CD133 positive rates in CNE‐2R and CNE‐2 cells were (2.49 ± 0.56%) vs (0.76 ± 0.25%; *P *=* *0.008), respectively. Meanwhile, the proliferation capacity, tumorigenesis capacity, and telomerase activity of CNE‐2R‐CD133+ cells were notably higher than those of CNE‐2R‐CD133− and CNE‐2R cells. Collectively, CNE‐2R displayed CSC‐like characteristics; our results also showed that CNE‐2R cells, especially the sorted CSCs, had high telomerase activity levels.

## 1 INTRODUCTION

Radiotherapy is a major treatment and an essential component of curative treatment for nasopharyngeal carcinoma (NPC).^1^ As radiotherapy technology and the therapeutic scheme are consistently updated, the 5‐year overall survival (OS) rate of NPC has reached approximately 80%.^2^, ^3^ Nonetheless, over 20% of NPC patients develop recurrence or distant metastasis after standard treatment.^3^, ^4^, ^5^ Radioresistance is often regarded as an important cause of failure in NPC treatment.^6^, ^7^ Currently, the potential mechanism of radioresistance in NPC remains unknown. Previous studies have extensively explored the radiosensitivity and radioresistance mechanisms of NPC from multiple angles, such as hypoxia in cancer cells, DNA damage repair, and gene regulation, but the underlying problem has not been resolved.^8^

In recent years, some scholars hypothesized that the resistance, recurrence, and metastasis of cancer might be related to cancer stem cells (CSCs).^9^, ^10^, ^11^, ^12^, ^13^ CSCs have been considered one of the few cancer cells that have out‐of‐control proliferation characteristics, including self‐renewal, multipotential differentiation, and stem cell characteristics. Some studies have indicated that CSCs are related to the radioresistance of cancers.^14^, ^15^, ^16^, ^17^, ^18^, ^19^ For example, Krause et al suggested that CSCs could mediate the occurrence of radioresistance in cancer through multiple mechanisms.^16^, ^17^ In addition, NPC‐related studies also found that CSC‐like cancer cells had marked radioresistant characteristics.^20^, ^21^, ^22^, ^23^ However, previous studies focused on exploring the biological characteristics and the radioresistance of NPC CSCs, while few studies have examined whether NPC radioresistant cells have CSC‐like characteristics. Existing studies have reported that radioresistant esophageal carcinoma cells exhibit more remarkable CSC‐like characteristics than parental radiosensitive cells.^24^, ^25^ Su et al^26^ found that ionizing radiation could activate the CSC phenotype of nasopharyngeal carcinoma cells. Therefore, we speculate that NPC radioresistant cells also have CSC‐like characteristics.

Telomerase is a ribonucleoprotein enzyme complex that mainly plays a role in adding the 5′‐TTAGGG‐3′ repeated sequence into the terminal end of human chromosomes and participates in the telomerase maintenance mechanism of approximately 90% of human cancers.^27^ Telomerase is markedly expressed in CSCs and, as such, is speculated to be associated with CSCs.^28^ Ju et al^29^ reported that telomerase was necessary for the progression, self‐renewal, and immortalization of CSCs. Zhang et al^25^ discovered that esophageal cancer radioresistant cells had more significant telomerase activity than that of parental radiosensitive cells. In addition, previous studies also indicated that the positive rate of telomerase in NPC was approximately 84%.^30^ In conclusion, we speculated that NPC radioresistant cells would have more telomerase activity. This paper explores the CSC‐like characteristics and telomerase activity of NPC radioresistant cells, as well as the correlation between the two, to provide a foundation for studies on CSCs targeted therapy for radioresistant NPC.

## 2 MATERIALS AND METHODS

### 2.1 Cell lines and cell culture

CNE‐2, a human nasopharyngeal carcinoma poorly differentiated cell line, was purchased from cancer Hospital of Fudan University (Shanghai, China). The cell line has been tested through DNA (STR) profiling on 15 March 2018. We established a radioresistant cell lines (CNE‐2R) by subjecting the CNE‐2 to fractionated radiation in previous study.^31^ Cells were cultured in DMEM medium containing 10% fetal bovine serum (FBS; Gibco, Grand Island, New York, USA), 100 U/mL penicillin, and 0.1 mg/mL streptomycin (Solarbio, Beijing, China) at 5% CO_2_ and 37°C in a saturated humidity incubator (Heal Force, Shanghai, China).

### 2.2 Radioresistance of CNE‐2R detected using a clone formation assay

CNE‐2R and CNE‐2 cells were seeded onto 6‐well plates at a density of 200, 200, 400, 600, and 1000 in a saturated humidity incubator at 5% CO_2_ and 37°C. After cell adherence, the cells were treated with 6 MV‐X‐ray radiation at various doses (0, 2, 4, 6, and 8 Gy for each cell density). The cells were then cultured for 14 days, fixed with 4% paraformaldehyde (Solarbio) for 30 minutes, and stained with Giemsa solution (Solarbio). The clone numbers that formed at each radiation dose in two groups were counted, and the colonies with ≥50 cells were deemed an effective clone. The dose‐survival curve was fitted using the single‐hit, multitarget model *y* = 1−(1−exp (−k*x))^*N*^, where *D*_0_ = 1/*K* and *D*_q_ = lnN*D_0_.

### 2.3 Reverse transcription and real‐time quantitative PCR (qPCR)

RNA was extracted using TRIzol (Invitrogen, Carlsbad, California, USA), and cDNA was obtained through reverse transcription using the PrimeScript RT reagent Kit (Takara Bio, Kusatsu, Shiga, Japan). qPCR was carried out using SYBR Premix Ex TaqTM II (Takara Bio) to detect the mRNA expression of stem cell‐related genes, the telomerase reverse transcriptase (hTERT) gene, and GAPDH as the endogenous reference gene. The primers used were as follows: (5′→3′): Oct4‐F: GCTGGATGTCAGGGCTCTTTG, Oct4‐R: TTCAAGAGATTTATCGAGCACCTTC; Sox2‐F: GTGAGCGCCCTGCAGTACAA, Sox2‐R: GCGAGTAGGACATGCTGTAGGTG; Nanog‐F: CCTGTGATTTGTGGGCCTGA, Nanog‐R: CTCTGCAGAAGTGGGTTGTTTG; Bmi1‐F: AAATGCTGGAGAACTGGAAAG, Bmi1‐R: CTGTGGATGAGGAGACTGC; CD133‐F: AGTGGCATCGTGCAAACCTG, CD133‐R: AGTGGCATCGTGCAAACCTG; hTERT‐F: CTCCCATTTCATCAGCAAGTTT, hTERT‐R: CTTGGCTTTCAGGATGGAGTAG; GAPDH‐F: CAGGAGGCATTGCTGATGAT, GAPDH‐R: GAAGGCTGGGGCTCATTT.

### 2.4 Protein expression detected by Western blot

The total protein content of CNE‐2R and CNE‐2 cells was extracted, 50 μg of total protein was taken for electrophoresis, and the electrophoresis products were transferred onto polyvinylidene fluoride (PVDF) membranes (Millipore, Billerica, Massachusetts, USA) using the wet transfer method. The PVDF membranes were blocked with 5% skim milk for 1.5 hours, washed with TBST, incubated with primary antibodies, and then shaken at 4°C overnight. The PVDF membranes were then removed and washed with TBST. The membranes were then incubated with a secondary antibody (CST, Danvers, Massachusetts, USA) at room temperature for 1 hour prior to being washed with TBST. Images were collected using the Gel Imaging System (Bio‐Rad, Hercules, California, USA). Primary antibodies Oct4, Sox2, Nanog, Bmi1, β‐catenin, and β‐actin were purchased from Cell Signaling Technology (CST), while hTERT was purchased from Abcam Company (Abcam, Cambridge, UK).

### 2.5 Label‐retaining cells (LRCs) detected by immunocytochemistry

Cells were cultured in complete medium containing 10 μmol/L of 5‐bromodeoxyuridine (BrdU; Sigma, St. Louis, Missouri, USA) for 7 days consecutively. Then, the BrdU was removed, and the cells were cultured for another 14 days. The cells climbing to the coverslips were conducted before the addition of BrdU, as well as on day 7 after adding BrdU and day 14 after removing the BrdU. The coverslips were removed after cell adherence and fixed with 4% paraformaldehyde. Endogenous peroxidase activity was blocked with 3% H_2_O_2_. DNA denaturation was carried out with 2 mol/L HCl. Enzymatic pretreatment was performed with 0.1% trypsin. Nonspecific antigens were blocked with 3% BSA. Mouse monoclonal anti‐BrdU (dilution: 1:1000; Sigma) was applied overnight at 4°C, followed by a biotinylated secondary antibody and HRP‐labeled streptavidin using the mouse SPlink Detection Kit (ZSGB‐BIO, Beijing, China). The peroxidase reaction was developed using the DAB kit (ZSGB‐BIO). The cells were then counterstained with hematoxylin. A total of 10 high‐power fields (200×) were randomly selected under the light microscope (Olympus, Tokyo, Japan) to calculate a proportion of positive cells. This experiment was repeated three times.

### 2.6 Telomerase activity detected by PCR‐ELISA

Telomerase activity was detected with the TeloTAGGG Telomerase PCR‐ELISA kit (Roche, Basel, Switzerland) according to manufacturer's instructions. The cells were harvested, and 2 × 10^5^ cells were transferred into a reaction tube. The cells were suspended in 200 μL lysis reagent and incubated on ice for 30 minutes. The lysate was centrifuged at 16 000 *g* for 20 minutes at 4°C. Next, 175 μL supernatant was collected (cell extract), and then 2 μL cell extract (corresponding to 2 × 10^3^ cell equivalents) and 25 μL reaction mixture were added to a tube with sterile water to bring the final volume to 50 μL for PCR amplification. Then, 5 μL of the amplification product and 20 μL of the denaturation reagent were added to a tube and incubated for 10 minutes at 20°C. Next, 225 μL hybridization buffer was added per tube and mixed thoroughly. A total of 100 μL of the mixture was transferred to each well of the MP modules supplied with the kit prior to incubation at 37°C for 2 hours. The hybridization solution was removed and washed with washing buffer. A total of 100 μL anti‐DIG‐POD working solution was added per well and incubated at 20°C for 30 minutes. The solution was removed and rinsed with washing buffer. Then, 100 μL TMB substrate solution was added per well and incubated at 20°C for 15 minutes. A total of 100 μL stop reagent was then added per well, without removing the reacted substrate. Using a microplate reader (Thermo, USA), absorbance was measured at 450 nm (with a reference wavelength of 690 nm) within 30 minutes after the addition of the stop reagent. The 293 cell extract was used as a positive control, and the RNase‐treated extract was used as a negative control. This experiment was performed in triplicate and repeated three times.

### 2.7 Flow cytometry (FCM) and magnetic‐activated cell sorting (MACS)

A total of 1 × 10^7^ cells was harvested and suspended in 100 μL of buffer. Then, 10 μL mouse CD133‐PE antibody (Miltenyi Biotec, Teterow, Germany) was added and incubated in the dark at 4°C for 10 minutes. The cells were washed twice with buffer and suspended in 500 μL of buffer for analysis by flow cytometry (BD FACSCalibur, San Jose, California, USA). A mouse IgG1 isotype antibody (Miltenyi Biotec) was used as the control. This experiment was repeated three times. We used the CD133 MicroBead Kit (Miltenyi Biotec) for cell sorting. A total of 1 × 10^7^ cells was harvested and suspended in 60 μL of buffer prior to the addition of 20 μL FcR blocking reagent and 20 μL CD133 MicroBeads, and the mixture was incubated for 10 minutes at 4°C. The cells were washed twice with buffer and then suspended in 500 μL of buffer. Next, magnetic separation was performed according to the manufacturer's instructions. Unlabeled cells passed through, while labeled cells were retained in the column. Labeled and unlabeled cells were separately collected for further experiments.

### 2.8 CCK‐8 assay and sphere formation assay

Cell viability was detected with the CCK‐8 assay kit (Dojindo, Tokyo, Japan). Cells were plated in 96‐well plates at a density of 2 × 10^3^ cells per well. After culturing for 0, 24, 48, 72, 96, and 120 hours, removed the culture medium, and added 100 μL fresh medium and 10 μL CCK‐8 reagent into each well and cultured at 37°C for 1 hour. The absorbance was measured using a microplate reader at 450 nm. Each experiment was performed in triplicate and repeated three times.

Sphere formation assay was used to identify CSCs. Single cells (2 × 10^3^) were seeded onto the 6‐well ultralow attachment plate (Corning, New York, USA) in serum‐free DMEM‐F12 (Gibco), supplemented with 20 ng/mL EGF, 20 ng/mL bFGF, 4 μg/mL insulin, and 2% B27 (Sigma). Sphere formation was observed under the inverted light microscope (Olympus) everyday, and the formed sphere number was counted under the microscope after 9 days of culture.

### 2.9 Tumorigenesis in vivo experiment

BALB/C nude mice (4‐6 weeks old) were purchased from the Laboratory Animal Center of Guangxi Medical University. CNE‐2R‐CD133+, CNE‐2R‐CD133−, and CNE‐2R cells were subcutaneously injected into the right groin at doses of 5 × 10^3^, 10^4^, and 10^5^, respectively. Five nude mice were assigned to each group, with 45 nude mice in total. Tumorigenesis in nude mice was observed within 4 weeks, and the tumorigenesis rate was calculated. The procedures involving animals and their care were approved by Laboratory Animal Care and Use Committee of the Guangxi Medical University.

### 2.10 Statistical analysis

Statistical analysis was conducted using SPSS 20.0 (IBM, Armonk, NY, USA) or GraphPad Prism 5.0 (GraphPad Software, San Diego, CA, USA) software. The experimental data were expressed as the mean ± standard deviation (SD). Intergroup differences were compared with a two‐tailed Student's *t* test or a one‐way ANOVA. A two‐tailed difference of *P *<* *0.05 was deemed statistically significant.

## 3 RESULTS

### 3.1 CNE‐2R cells displayed obvious radioresistance

**Figure 1:**
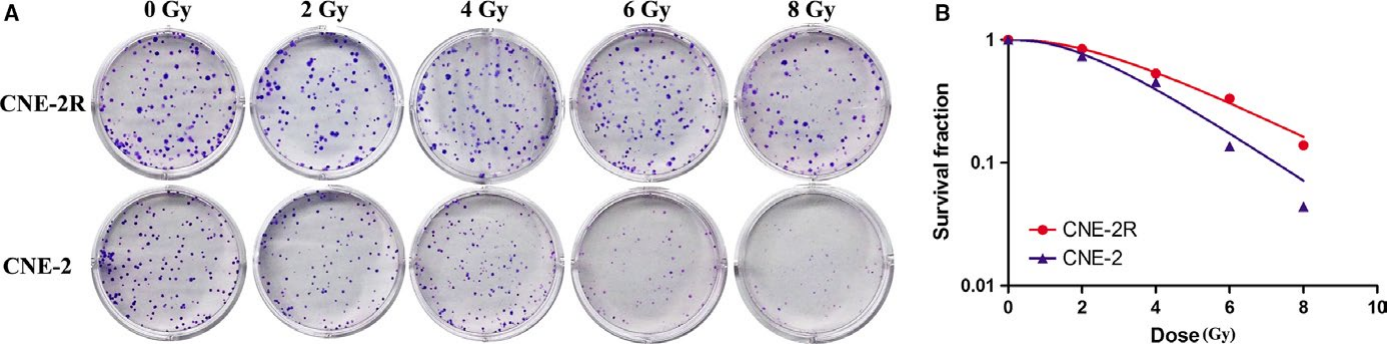
CNE‐2R cells displayed obvious radioresistance. A, Radiosensitivity of CNE‐2R and CNE‐2 cells compared through a clone formation assay. B, The dose‐survival curve fitted using the single‐hit multitarget model

The radiosensitivity of CNE‐2R and CNE‐2 cells was compared with a clone formation assay (Figure 1A). The dose‐survival curve was fitted using a single ‐hit, multitarget model that suggested that the survival fractions of CNE‐2R cells at all radiation doses were markedly higher than those in CNE‐2 cells (Figure 1B). The radiobiological parameters are shown in Table 1. Compared with CNE‐2 cells, the sensitizing enhancement ratio (SER) of CNE‐2R cells = *D*_0_ (CNE‐2)/*D*_0_ (CNE‐2R) = 0.67 < 1 revealed that CNE‐2R cells had radioresistance.

**Table 1:** Radiobiological parameters of CNE‐2R and CNE‐2 cells (Mean ± SD)

Cell lines	D_0_	*D*_q_	SF_2_
CNE‐2R	3.005 ± 0.222	2.751 ± 0.336	0.834 ± 0.033
CNE‐2	2.286 ± 0.112	2.001 ± 0.274	0.701 ± 0.037
*P*	0.007	0.040	0.010

*D*_0_ was the mean lethal dose, which was theoretically the radiation dose required to hit each cell; *D*_q_ was the quasi‐threshold dose, which reflected the repair capacity of sublethal cell injury; and SF_2_ was the survival fraction at the dose of 2 Gy.

### 3.2 Identification of CSC‐like characteristics of CNE‐2R cells

**Figure 2:**
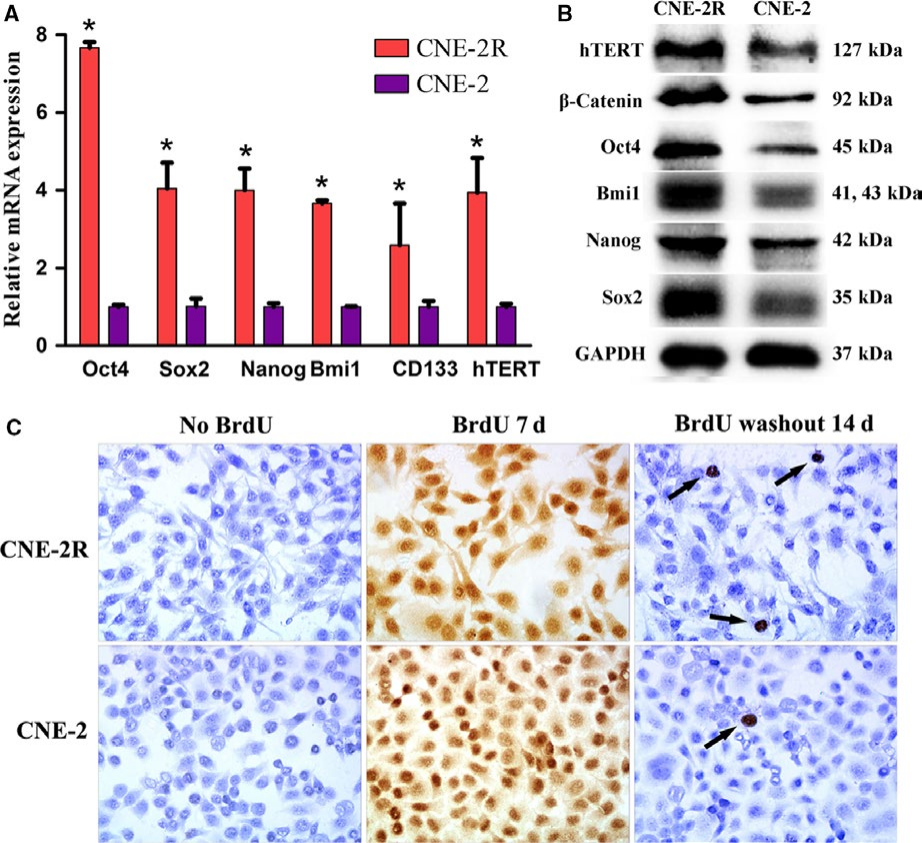
CSC‐like characteristics identification in CNE‐2R cells. A, Expression levels of stem cell‐related genes and hTERT gene in CNE‐2R cells were notably higher than those in CNE‐2 cells (* indicated value compared with CNE‐2 with *P *<* *0.05); B, Stem cell‐related proteins and hTERT protein expression level in CNE‐2R cells were markedly higher than that in CNE‐2 cells; C, LRCs’ proportion in CNE‐2R cells was higher than that in CNE‐2 cells (200×; *P *<* *0.001). Black arrows indicated LRCs

To preliminarily determine whether CNE‐2R cells exhibited CSC‐like characteristics, we detected the expression levels of some stem cell‐related genes and the hTERT gene using qPCR. These results indicated that the mRNA expression levels of stem cell‐related genes (Oct4, Sox2, Nanog, Bmi1 and CD133) and the hTERT gene in CNE‐2R cells were remarkably higher than those in CNE‐2 cells (*P *<* *0.001; Figure 2A). In addition, the results from the Western blot suggested that the protein expression of Oct4, Sox2, Nanog, Bmi1, β‐catenin, and hTERT in CNE‐2R cells was dramatically higher than that in CNE‐2 cells (Figure 2B).

The LRCs’ proportions were detected with immunocytochemistry as a means to further verify the CSC‐like characteristics of CNE‐2R cells. These results suggested that no positive cells were observed in both CNE‐2R and CNE‐2 cells before the addition of BrdU, but nearly 100% positive cells were observed on day 7 after the addition of BrdU; after the removal of BrdU, only a few positive cells were observed on day 14, and these were the LRCs (Figure 2C). The LRC proportions in CNE‐2R and CNE‐2 groups were (3.10 ± 0.63%) vs (0.40 ± 0.35%), (*P *<* *0.001).

### 3.3 CNE‐2R cells contained a higher proportion of CD133‐positive cells

**Figure 3:**
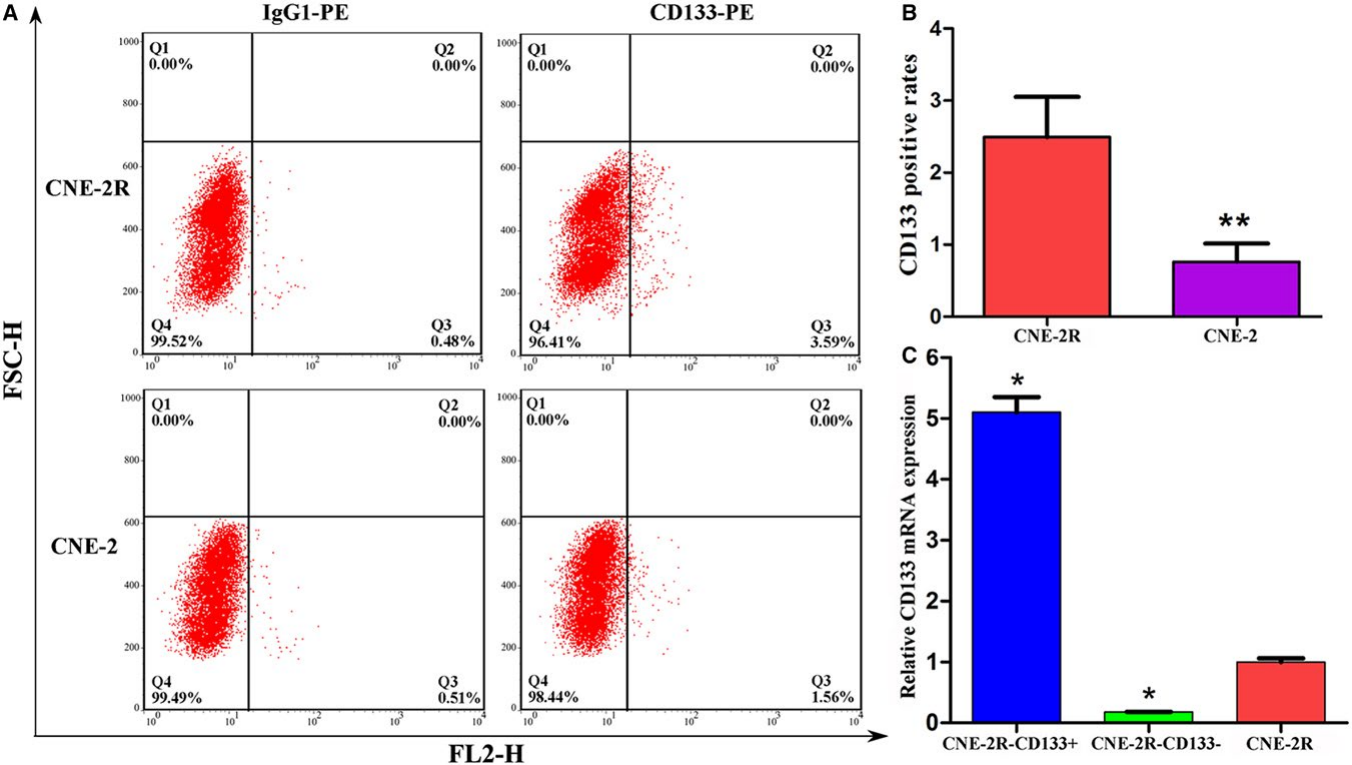
CD133 expression in CNE‐2R cells was higher than that in CNE‐2 cells. A, CD133 expression rates in CNE‐2R and CNE‐2 cells detected using FCM; B, quantification of CD133 positive rates (** indicated value compared with CNE‐2R with *P *<* *0.01); C, CD133 mRNA expression in CNE‐2R‐CD133+ cells was dramatically higher than that in CNE‐2R‐CD133− and CNE‐2R cells (* indicated value compared with CNE‐2R with *P *<* *0.05)

The expression of the stem cell marker CD133 was further determined through FCM in both groups of cells, and the results showed that the CD133 expression in CNE‐2R cells was markedly higher than that in CNE‐2 cells (Figure 3A), and the CD133 expression rates were (2.49 ± 0.56%) vs (0.76 ± 0.25%), respectively, (*P *=* *0.008, Figure 3B).

CNE‐2R‐CD133+ and CNE‐2R‐CD133− cells were obtained through MACS. The number of CNE‐2R‐CD133+ cells after sorting was limited (approximately 3 × 10^5^) and could not meet the number of cells required for FCM. Therefore, the sorting effect was evaluated through qPCR. These results suggested that the mRNA expression level of CD133 in CNE‐2R‐CD133+ cells was higher than that in CNE‐2R‐CD133− and CNE‐2R cells (Figure 3C).

### 3.4 CNE‐2R‐CD133+ cells displayed strong self‐renewal and a high tumorigenesis capacity

**Figure 4:**
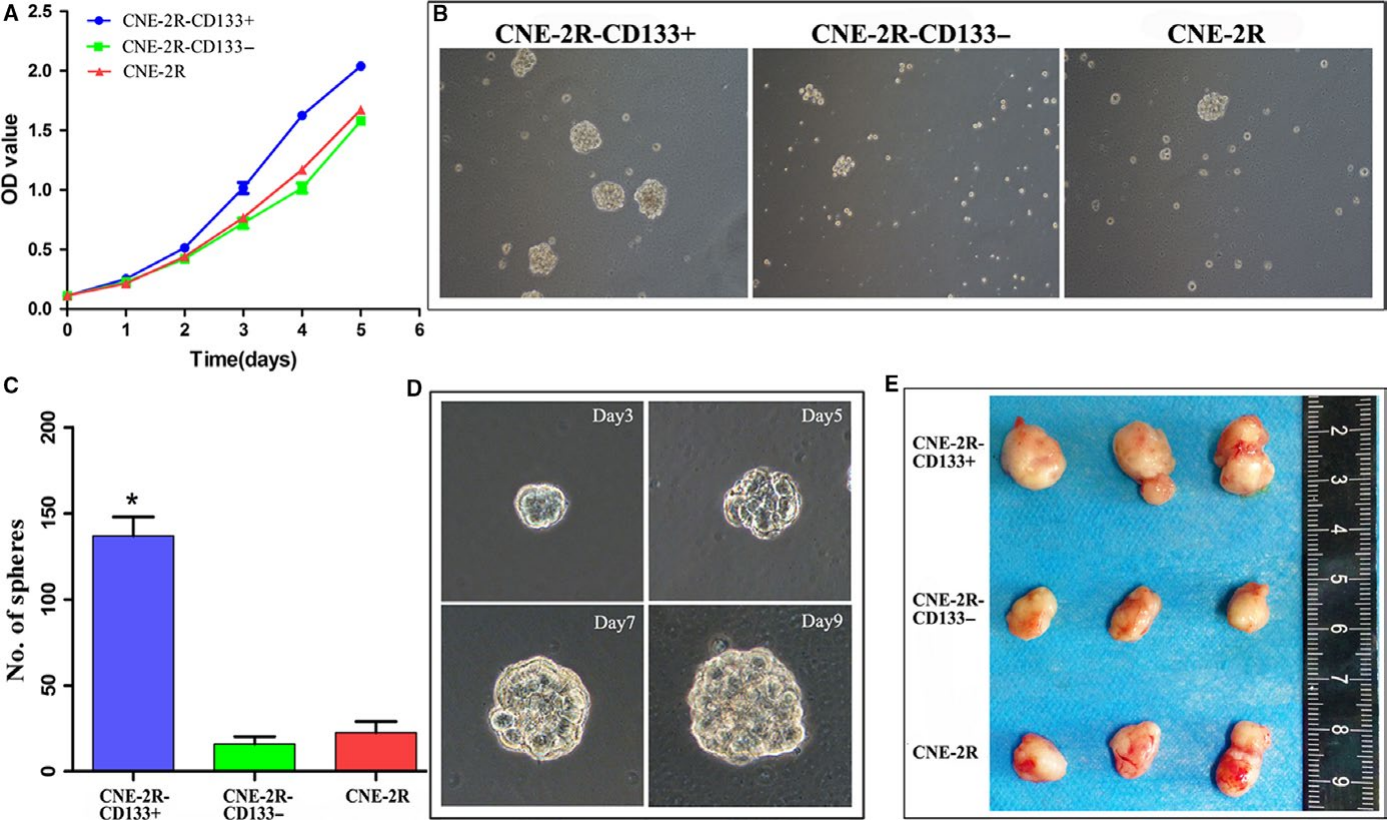
CNE‐2R‐CD133+ cells displayed CSCs’ characteristics both in vivo and in vitro. A, Growth curves of CNE‐2R‐CD133+, CNE‐2R‐CD133−, and CNE‐2R cells detected using CCK8 assay. B, Sphere formation of three groups of cells (100×); C, sphere number of three groups 9 days after culture (* indicated value compared with CNE‐2R with *P *<* *0.05); D, growth of spheres formed by CNE‐2R‐CD133+ cells (200×); E, tumor size in three groups after injection with 1 × 10^5^ cells

The in vitro biological characteristics of CNE‐2R‐CD133+ cells were determined by performing a CCK‐8 assay and a sphere formation assay. The results of the CCK‐8 assay showed that CNE‐2R‐CD133+ cells grew more rapidly compared to CNE‐2R‐CD133− and unsorted CNE‐2R cells (Figure 4A). The results of the sphere formation assay indicated that the formed sphere number of CNE‐2R‐CD133+ cells was markedly higher than that of CNE‐2R‐CD133− and CNE‐2R cells (Figure 4B,C). In addition, the sphere size formed by CNE‐2R‐CD133+ cells was larger with the increase in culture time (Figure 4D).

The in vivo tumorigenesis capacity of CNE‐2R‐CD133+ cells was evaluated with an experiment using nude mice. The results revealed that 5 × 10^3^ CNE‐2R‐CD133+ cells could form tumors in nude mice (4/5), whereas 1 × 10^4^ CNE‐2R‐CD133− cells only led to the formation of tumors in one nude mouse (1/5; Table 2). Additionally, we discovered that the tumors formed by 1 × 10^5^ CNE‐2R‐CD133+ cells were noticeably larger compared to CNE‐2R‐CD133− and CNE‐2R cells (0.889 ± 0.016 cm^3^ vs 0.236 ± 0.030 cm^3^, 0.368 ± 0.016 cm^3^), (*P *<* *0.001; Figure 4E). These results demonstrated that CNE‐2R‐CD133+ cells exhibit increased proliferation, self‐renewal, and tumorigenesis capacities; thus, these cells display characteristics of CSCs.

**Table 2:** Tumorigenesis of CNE‐2R‐CD133+, CNE‐2R‐CD133−, and CNE‐2R cells in nude mice after 4 wk by subcutaneous injection

No. of injected cells	No. of tumor formed/injected		
	5 × 10^3^	1 × 10^4^	1 × 10^5^
CNE‐2R‐CD133+	4/5	5/5	5/5
CNE‐2R‐CD133−	0/5	1/5	3/5
CNE‐2R	0/5	2/5	4/5

### 3.5 The identification of telomerase activity in each group of cells

**Figure 5:**
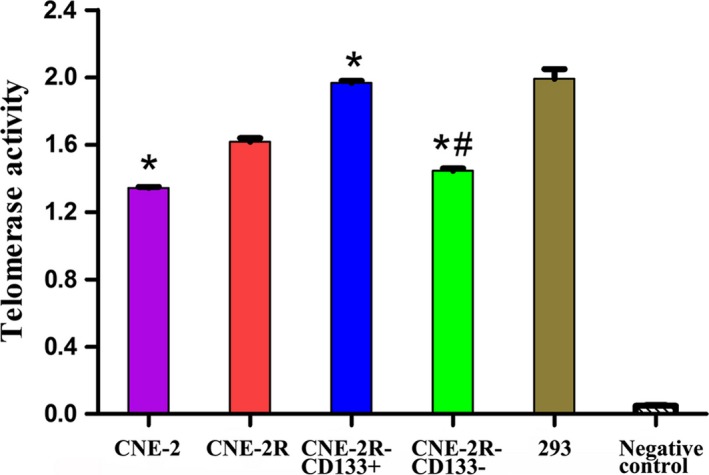
Telomerase activity of each group compared by PCR‐ELISA assay (* indicated value compared with CNE‐2R with *P *<* *0.05, and # indicated value compared with CNE‐2R‐CD133+ group with *P *<* *0.05)

Telomerase activity was detected using a PCR‐ELISA assay. These results indicated that the telomerase activity of CNE‐2R cells was higher than that in CNE‐2 cells (*P *<* *0.05). Similarly, the telomerase activity in CNE‐2R‐CD133+ cells was markedly higher than that in CNE‐2R‐CD133− and CNE‐2R cells (*P *<* *0.05; Figure 5).

## 4 DISCUSSION

The CSCs’ researches not only aid in the identification of problems associated with tumorigenesis, development, metastasis, and regeneration capacities but also contribute to illustrating the mechanisms by which tumors resist traditional treatment approaches. In 2006, the American Association for Cancer Research (AACR) proposed that CSCs may be novel targets for overcoming tumor drug resistance and possibly improve the efficacy of treatments and even prevent tumor formation.^32^ NPC CSC‐related studies have been previously reported in succession,^20^, ^21^, ^22^, ^23^, ^33^, ^34^ but few studies have explored whether NPC radioresistant cells exhibit CSC characteristics. We previously and successfully constructed the radioresistant cell line CNE‐2R through fractionated radiation.^31^ In this study, we aimed to determine whether CNE‐2R cells exhibit CSC‐like biological characteristics and to preliminarily explore their potential association with telomerase activity.

Other studies have verified that numerous types of CSCs and embryonic stem cells (ESCs) share similar gene expression patterns.^35^ Additionally, some ESC‐related core transcription factors and molecular markers were highly expressed in CSCs.^36^, ^37^ Our results indicated that the expression levels of some stem cell‐related genes and proteins (Oct4, Sox2, Nanog, Bmi1 and CD133) in CNE‐2R cells were distinctly higher than those in CNE‐2 cells; this finding suggests that CNE‐2R cells may possess CSCs’ characteristics. β‐catenin is one of the essential components involved in the Wnt signaling pathway. Previous studies have uncovered an association between high β‐catenin expression levels and the self‐renewal capacity of CSCs, which is related to the in vivo survival and tumorigenesis of CSCs.^38^, ^39^ The dynamic balance of β‐catenin between the cytoplasm and the nucleus is destroyed, while persistent accumulation will signal normal stem cells to transform into infinitely proliferating CSCs, thus forming a tumor; this process may also enhance the radio‐ and chemoresistant properties of CSCs.^40^, ^41^ NPC‐related research also indicates that β‐catenin plays a crucial role in maintaining the CSC phenotype, while verifying that the Wnt/β‐catenin pathway plays a regulatory role in the NPC of CSCs.^42^, ^43^ In this study, our Western blotting results suggested that the protein expression levels of β‐catenin in CNE‐2R cells were dramatically higher than that in CNE‐2 cells; these results further verify the CSC‐like characteristics of CNE‐2R cells.

BrdU is the brominated analog of thymidine and can selectively incorporate into DNA during the S phase of the cell cycle.^44^ In ordinary cells, BrdU gradually dilutes during cell proliferation and passage. However, stem cells belong to a group of slow cycle cells that possess an immortalized DNA strand that can be labeled by BrdU for an extended period. These cells are called LRCs, and the presence of CSCs can be detected through the identification of LRCs.^45^ Some studies have verified the presence of CSCs in NPC by identifying the presence of LRCs.^45^, ^46^ This study detected the presence of LRCs in CNE‐2R and CNE‐2 cells with immunocytochemistry, and we show that the proportion of LRCs in CNE‐2R cells is markedly higher than that in parental CNE‐2 cells. Thus, we verified with additional experiments that radioresistant CNE‐2R cells exhibit CSC‐like characteristics. Consequently, this result further supports studies on the CSC‐like characteristics of NPC radioresistant cells, as this may be a new and exciting avenue for improving radiosensitivity in NPC.

Additionally, we also showed that hTERT gene and protein expression levels in CNE‐2R cells were higher than those in CNE‐2 cells. hTERT is the essential catalytic subunit for maintaining telomerase activity; hTERT can regulate telomerase activity at the transcription level, and its expression level is consistent with telomerase activity.^47^ We examined telomerase activity in CNE‐2R and CNE‐2 cells, and the results definitely suggested that CNE‐2R cells exhibited increased telomerase activity. To further explore whether the high telomerase activity in CNE‐2R cells was induced by their CSC‐like characteristics, we separated and detected the biological characteristics of subgroups of CNE‐2R cells using the CD133 molecular marker with MACS. CD133 has been suggested as a molecular marker of CSCs in multiple tumor studies including NPC.^48^, ^49^, ^50^, ^51^, ^52^ Our results also suggested that CNE‐2R‐CD133+ cells had a strong in vitro proliferation capacity and in vivo tumorigenesis capacity, thus preliminarily verifying that CNE‐2R‐CD133+ cells are CSCs. Subsequently, we examined the telomerase activity of CNE‐2R‐CD133+ and CNE‐2R‐CD133− cells, and our results indicated that CNE‐2R‐CD133+ cells had increased telomerase activity. This finding suggests that CSCs may have infinite proliferation potential to produce tumor cells. These results are consistent with previous studies.^25^, ^28^, ^29^ Therefore, we suggest that telomerase‐specific therapy may be a new treatment strategy for targeting CSCs specifically which has provided a new thinking for overcoming the radioresistance in NPC.

It currently remains unclear whether the CSC‐like characteristics of radioresistant cancer cells are induced by the self‐adaptive transformation of cancer cells during the radiation process or through CSC enrichment induced by radiation; the research results by Su et al^26^ support the former possibility, while the results of Che et al^24^ supported the latter. Although this study did not extensively explore this mechanism, our research has uncovered some interesting findings and may provide a foundation for future studies. For example, CSCs may be the potential targets in treating radioresistant NPC. It would be interesting to study mechanisms to induce death in CSCs using the following strategies: One is the targeted therapy of CSC‐related signaling pathways, such as the interference of the Wnt/β‐catenin pathway to block CSCs, and the other is the specific targeted therapy of telomerase, as the telomerase‐specific oncolytic adenovirus may preferentially replicate in CSCs with high telomerase activity and induce death.

In conclusion, this study verified that the NPC radioresistant cells CNE‐2R induced by fractionated radiation displayed CSC‐like characteristics. Additionally, CNE‐2R cells, especially the sorted CSCs, exhibited high telomerase activity. The related underlying mechanisms of action are yet to be elucidated, but this work provided the foundation for further research on CSC‐targeted therapy for radioresistant NPC.

## CONFLICT OF INTEREST

The authors have no conflict of interest.
